# Novel heterozygous *TREX1* mutation in a juvenile systemic lupus erythematosus patient with severe cutaneous involvement treated successfully with Jak-inhibitors: a case report

**DOI:** 10.3389/fimmu.2023.1288675

**Published:** 2023-12-06

**Authors:** Martina Rossano, Emilio Amleto Conti, Paola Bocca, Stefano Volpi, Antonio Mastrangelo, Riccardo Cavalli, Marco Gattorno, Francesca Minoia, Giovanni Filocamo

**Affiliations:** ^1^ Pediatric Immuno-Rheumatology Unit, Fondazione IRCSS Ca’ Granda Ospedale Maggiore Policlinico, Milan, Italy; ^2^ Center for Autoinflammatory Diseases and Immunodeficiencies, IRCCS Istituto Giannina Gaslini, Genoa, Italy; ^3^ DINOGMI, Università degli Studi di Genova, Genova, Italy; ^4^ Pediatric Nephrology, Dialysis, and Transplant Unit, Fondazione IRCCS Ca’ Granda Ospedale Maggiore Policlinico, Milan, Italy; ^5^ Unit of Pediatric Dermatology, Foundation IRCCS Ca’ Granda Ospedale Maggiore Policlinico, Milan, Italy

**Keywords:** systemic lupus erythematosus, Trex1, JAK-inhibitor, baricitinib, pediatrics, case report

## Abstract

Juvenile systemic lupus erythematosus (jSLE) is a complex inflammatory autoimmune disorder. In the last decades, genetic factors and activation pathways have been increasingly studied to understand their potential pathogenetic role better. Genetic and transcriptional abnormalities directly involved in the type I interferon (IFN) signaling cascade have been identified through family-based and genome-wide association studies. IFNs trigger signaling pathways that initiate gene transcription of IFN-stimulated genes through the activation of JAK1, TYK2, STAT1, and STAT2. Thus, the use of therapies that target the IFN pathway would represent a formidable advance in SLE. It is well known that JAK inhibitors have real potential for the treatment of rheumatic diseases, but their efficacy in the treatment of SLE remains to be elucidated. We report the case of a 13-year-old girl affected by jSLE, carrying a novel heterozygous missense variant on Three prime Repair EXonuclease 1 (*TREX1*), successfully treated with baricitinib on top of mofetil mycophenolate. The *TREX1* gene plays an important role in DNA damage repair, and its mutations have been associated with an overproduction of type 1 interferon. This report underlines the role of translational research in identifying potential pathogenetic pathways in rare diseases to optimize treatment.

## 1 Introduction

Juvenile-onset systemic lupus erythematosus (jSLE) is a multisystemic autoimmune disease characterized by a heterogeneous presentation (1). Although jSLE pathogenesis has not been fully understood, several elements, including genetic and environmental factors, have been increasingly recognized as playing a key role. High serum levels of interferon (INF)α have been described in patients with SLE (2) for many years, and, more recently, INF-stimulated genes (ISG) were found to be overexpressed in patients with SLE, confirming the pivotal role of INFs in its pathogenesis (2, 3). Overexpression of these genes, measured as mRNA transcripts in peripheral blood cells, is called an “IFN signature” (2). The presence of a distinctive IFN signature is a strong clue to the pathway involved in the development of SLE and is frequently observed in jSLE at onset or during a disease flare (2–5).

The dysregulation of type I IFN signaling might be related to several genetic factors (5). Type I interferonopathies, rare Mendelian autoinflammatory disorders, are monogenic diseases characterized by exacerbated type I IFN signaling activity and an elevated ISG signature (6). Several mutations have been identified as pathogenetic in type I interferonopathies (7). One of the proposed mechanisms underpinning these conditions involves the accumulation of DNA and RNA pools caused by loss-of-function mutations involved in degradation of nucleic acids. In Aicardi-Goutières syndrome, pathogenetic mutations in the Three prime Repair EXonuclease 1 (TREX1) gene, which encodes a nuclear protein with 3’exonuclease activity (2), cause defective clearance of chromatin DNA, increased INF production, and an immune-mediated inflammatory response (8, 9).

The pathogenic role of type I IFN dysregulation in SLE, together with the growing evidence regarding the efficacy of JAK-inhibition in monogenic interferonopathies, advocates for the use of therapy to neutralize the activation of this particular pathway by blocking downstream JAK/STAT signaling in SLE (2). JAK- inhibitors (JAKi) are oral small molecules that block JAK–STAT signaling. Although JAKi are currently approved for the treatment of rheumatoid arthritis, polyarticular juvenile idiopathic arthritis, and severe atopic dermatitis, their potential role in the treatment of SLE still needs to be tested (2–4).

In this report, we described the case of a jSLE girl with an extremely severe and refractory skin involvement carrying a novel heterozygous missense mutation in *TREX1* who was successfully treated with the JAK 1/2 inhibitor baricitinib.

## 2 Case report

A 13-year-old girl adopted from Cambodia presented with malar rash, leukopenia (WBC 2620/mmc, L 1130/mm3, N 1130/mm3), hypocomplementemia C3 75 mg/dl (90-180 n.v.), C4 8 (10-40 mg/dl n.v.), hypergammaglobulinemia, increased level of ESR (49 mm/h 0-20 n.v.), positivity of anti-dsDNA (1278 UI/ml 0-27 n.v.), anti-Sm (693.5 UA 0-20 n.v.), and anti-SSA/Ro antibodies (1374.8 UA 0-20 n.v.). The diagnosis of jSLE was made, both ACR1997 (9) and SLICC-2012 (10) criteria were satisfied, and she was started on oral prednisone (2 mg/kg/day) and hydroxychloroquine. One year later, while she was on a low dose of oral prednisone (2.5 mg/day), a disease flare occurred, with hematological and dermatological involvement, requiring an increase of the daily prednisone dose up to 25 mg/day. However, further tapering of corticosteroids led to a new flare with severe vasculitic lesions mainly involving the face which are associated with leukopenia and increased acute phase reactants. The child was therefore started on mycophenolate mofetil (MMF) at a daily dose of 1000 mg.

**Figure 1:**
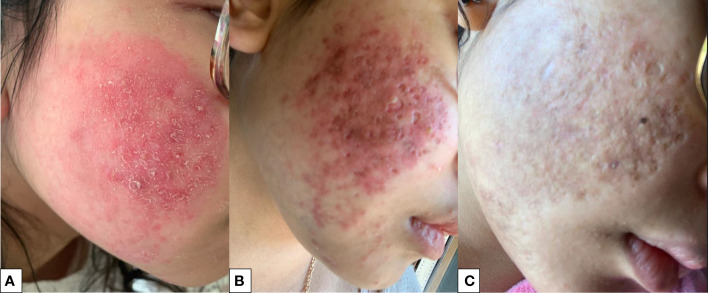
**(A)** Skin infiltrative lesions during treatment with steroids and MMF **(B)** skin infiltrative lesions right before treatment with baricitinib; **(C)** skin after 18 months of treatment with baricitinib.

Despite MMF treatment, each attempt to taper systemic steroids failed, and the patient experienced disease relapses, characterized by severe skin involvement with vasculitis, alopecia, and lymphopenia and elevation of anti-dsDNA, requiring again high doses of corticosteroids (50 mg/day) and an increase in the daily MMF dose up to 1500 mg/die. However, cutaneous involvement remained uncontrolled, with severe facial erythematous infiltrative plaques and vasculitic lesions, which are associated with stable high titers of anti ds DNA and elevation of ESR, despite the addition of a topical steroid (**Figure 1A**). Skin involvement was refractory to multiple therapeutic approaches during the following years, which included monthly infusions of intravenous immunoglobulin (IVIG) (14 total infusions) and association with tacrolimus (1.5 mg twice a day) or azathioprine (100 mg/day) with steroid dependence and corticosteroid toxicity (hypertrichosis, weight gain, irritability) (**Figure 1B**).

**Figure 2:**
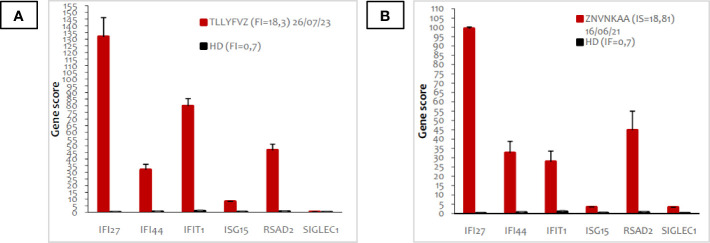
Peripheral blood type I interferon signature of the patient before **(A)** and after **(B)** treatment with baricitinib. The panel shows the expression of six types of I-IFN stimulated genes, indicated in the X axis. Red bars indicate expression levels in the patient; black bars represent the average of 20 healthy donors. The interferon score (FI) is indicated in the legend.

Given the peculiarity of the refractory skin involvement with severe vasculitis, a next-gene-sequencing (NGS) gene panel was performed, revealing a previously unreported heterozygous missense mutation on the *TREX1* gene (c.374A>G; p.Asn125Ser). Furthermore, the INF signature result was strongly positive (18,81 v.n. <0,7) (**Figure 2**). Assuming a potential role of the *TREX1*-related pathway in triggering disease flares, especially skin involvement, azathioprine was switched to the JAK 1/2 inhibitor baricitinib (2 mg three times a day).

Baricitinib led to a significant improvement of skin lesions, with a prompt reduction of erythema and cutaneous swelling after a few weeks. One month after azathioprine discontinuation, a persistent proteinuria was noted (proteinuria to creatininuria ratio 0.5). Renal biopsy showed a lupic nephritis IV stage, and MMF was re-started, in association with baricitinib, achieving a good renal response.

**Figure 3:**
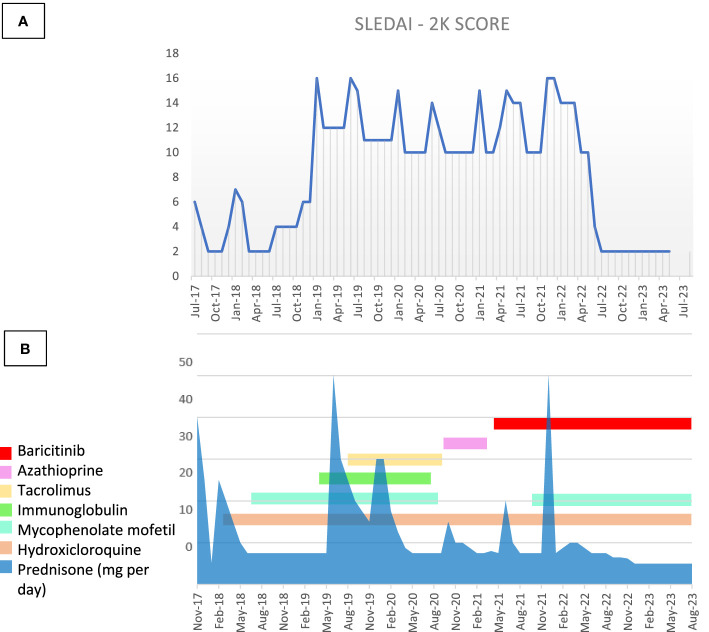
**(A)** SLEDAI-2K score and **(B)** therapy timeline through the disease course. Figure steroids and therapy changes over time in correlation with the modification of the SLEDAI-2K.

At the last visit, 18 months after starting baricitinib, optimal disease control has been obtained, especially in terms of cutaneous involvement (**Figure 1C**) (ERS 25 mm/h 0-20n.v., anti dsDNA 412.6 UI/ml 0-27 n.v.), together with a significant reduction in glucocorticoid use (daily prednisone 5 mg/day; previously, the daily dose of prednisone was at least 10 mg/day) without any adverse event or infection reported. **Figure 3** shows in details the patient’s treatment timeline, and in particular the daily steroidal dose, together with the SLEDAI-2k score over time.

## 3 Discussion

Over the past decades, the increased knowledge of pathways involved in disease pathogenesis has increased the relevance of precision medicine (10). Monogenic disorders may provide prototypes for pathogenesis-targeted therapies (11). So far, more than 80 loci associated with susceptibility in polygenic lupus have been identified, and screening for the activation of specific patterns can aid risk stratification of patients and optimization of treatment (12–14). JAKi have been proposed to be effective in blocking inflammation mediated by interferon overproduction (15). Accordingly, an increased INF signature score may identify potential responders to JAKi (12–14). In the present study, we report a patient with jSLE with refectory cutaneous involvement and a novel heterozygous missense mutation in *TREX1* who was successfully treated with baricitinib.

Despite several combinations of immunosuppressive treatments, our patient suffered from severe refractory skin involvement. The evidence of a strong positive INF signature together with the presence of a variant in the *TREX1* gene were crucial to support the introduction of a JAKi. The observed heterozygous *TREX-1* missense mutation (c.374A>G) replaces asparagine with serine at codon 125 of the TREX1 protein (p.Asn125Ser). The asparagine residue is highly conserved. This variant was not found in population databases nor in the dbSNP database. All algorithms developed to predict the effect of missense changes on protein structure and function (SIFT, PolyPhen-2, Align-GVGD) suggested a potential disruptive role of the variant reported. Unfortunately, the unavailability of the biological parents prevented further studies on the role of the detected variant in the family. Despite the lack of functional tests to prove our hypothesis, its rarity, in silico pathogenic prediction, clinical presentation, and impressive clinical response to JAK-inhibition highly support its pathogenetic contribution.

Indeed, *TREX1* mutations have been observed with increased frequency (0.5–3%) in SLE patients compared to healthy controls (16, 17). Furthermore, deficient TREX1 activity has been related to severe cutaneous involvement both in Aicardi-Goutieres syndrome and familial chilblain lupus, suggesting skin is a target tissue of the TREX1-related pathway (17, 18).

Baricitinib is a first-generation JAKi, and it inhibits all four members of the JAK family (JAK1, JAK2, JAK3, and TYK2) with a preferential action on JAK1 and JAK2 (19, 20). After several clinical trials, JAKi have been increasingly used for adults with connective tissue disease including SLE. In the pediatric population, many case series reported the beneficial use of JAKi (20–22), especially in children with monogenic interferonopathies (17–23). Baricitinib was chosen over other JAK inhibitors due to the safety and efficacy shown in pediatric trials on JIA and SAVI syndrome (23, 24).

However, randomized controlled trials in children with jSLE to properly examine the efficacy and safety of JAK inhibitors, also in combination with disease-modifying anti rheumatic drugs (DMARDs), are lacking (25), and most of the existing data about JAKi safety are derived from adult cohorts or pre-clinical studies (26–29). Several case series supported the beneficial effect of JAKi in pediatric patients with interferonopathies caused by *TREX1* mutations (12–22) and the most frequent adverse event reported was the increased risk of viral infections due to the suppression of INF signaling (15–19).

During the follow-up, we closely monitored clinical and laboratory parameters of disease activity and viral infections, in particular EBV, CMV, and polyomavirus, along with the IFN signature. This particular parameter was reassessed after 18 months of treatment, revealing stable results. In existing literature, the role of IFN-signature as a biomarker of treatment response remains ambiguous, with documented fluctuations observed throughout the day and across different follow-up timelines (17, 30). In a multicenter study that evaluates patients with monogenic interferonopathies, the IFN signature was normalized only in 5 patients with CANDLE out of the 10 who achieved clinical remission (17).

To our knowledge, this is the first report of a jSLE patient with refractory cutaneous involvement carrying a mutation in the TREX1 gene who was successfully treated with a JAKi.

In 2018, a phase II trial evaluating baricitinib in adult patients with SLE proved that a 4 mg/daily dose significantly improved signs and symptoms of active disease in patients who were not adequately controlled by standard of care therapy, with a good safety profile. However, authors did not find a significance difference between baricitinib and a placebo in terms of efficacy on mucocutaneous involvement (31). Although a first phase III trial (SLE-BRAVE-I) confirmed the positive results of phase II (32), those were not replicated by a second phase III trial (SLE-BRAVE-II) (33, 34). A Phase I study found tofacitinib to be safe in SLE and to improve cardiometabolic and immunologic parameters (35), and a second phase I/II trial testing tofacitinib in adult SLE with moderate and severe skin involvement is currently ongoing (NCT03288324). Note that an ongoing clinical trial is evaluating the expression of JAK3 in blood and renal tissue during active disease in children with jSLE (NCT04293510). Results from the latter two studies may further support the use of targeted JAK-inhibition for pediatric lupus patients (20).

In conclusion, our study suggests that the p.Asn125Ser might represent a novel pathogenic *TREX1* gene mutation linked to a jSLE phenotype. The impressive clinical response to JAK inhibition in our patient highlights the importance of including genetic analysis in refractory jSLE patients to foster a target treatment and optimization of care. Furthermore, our study supports the efficacy and safety of combination therapy with MMF and JAKi in a pediatric patient with jSLE with severe refractory cutaneous involvement.

## Data availability statement

The datasets presented in this article are not readily available because this is a Case Report.

## Ethics statement

The studies involving humans were approved by CET Lombardia 3. The studies were conducted in accordance with the local legislation and institutional requirements. Written informed consent for participation in this study was provided by the participants’ legal guardians/next of kin. Written informed consent was obtained from the individual(s), and minor(s)’ legal guardian/next of kin, for the publication of any potentially identifiable images or data included in this article.

## Author contributions

MR: Conceptualization, Writing – original draft, Writing – review & editing. EC: Writing – original draft. PB: Writing – review & editing. SV: Writing – review & editing. AM: Writing – review & editing. RC: Writing – review & editing. MG: Writing – review & editing. FM: Writing – original draft, Writing – review & editing. GF: Writing – original draft, Writing – review & editing.
